# The high-intensity interval training (HIIT) and curcumin supplementation can positively regulate the autophagy pathway in myocardial cells of STZ-induced diabetic rats

**DOI:** 10.1186/s13104-023-06295-1

**Published:** 2023-02-25

**Authors:** Samira Sadeghi, Maryam Delphan, Masoumeh Shams, Fataneh Esmaeili, Mahsa Shanaki-Bavarsad, Mehrnoosh Shanaki

**Affiliations:** 1 Department of Medical Laboratory Sciences, School of Allied Medical Science, Shahid Beheshti University of Medical Sciences, Tehran, Iran; 2 Department of Exercise Physiology, Faculty of Physical Education and Sport Sciences, Alzahra University, Tehran, Iran; 3 Department of Clinical Biochemistry, Faculty of Medicine, Tehran University of Medical Sciences, Tehran, Iran; 4 Department of Neurology, Memory and Aging Center, University of California San Francisco, San Francisco, CA USA

**Keywords:** Diabetic cardiomyopathy, Autophagy, High-intensity interval training (HIIT), Curcumin

## Abstract

**Objective:**

Targeting autophagy is a new therapeutic strategy for the complications of diabetes,such as diabetic cardiomyopathy (DCM). During diabetes, increased or insufficient autophagic activity causes aberrations in cellular homeostasis. Regarding the conflicting and unclear results regarding the effect of HIIT and curcumin supplementation on the expression of genes associated to autophagy, this study aimed to assess whether 4-week high-intensity interval training (HIIT) and curcumin supplementation are able to influence the expression of autophagy-related genes in myocardial cells of diabetic rats.

**Methods:**

In an experimental design, 24 male Wistar rats were randomly divided into 4 groups: non-diabetic control (NC), diabetic control (DC), diabetes + HIIT (D + HIIT), and diabetes + curcumin (D + CU). After HIIT program and curcumin treatment, the genes expression of autophagy pathway were assessed in the myocardium by real-time PCR Tanique.

**Results:**

The results indicated that the expression levels of ATG1, Beclin1, ATG5, and LAMP-2 genes were significantly reduced in the DC group compared to the NC group (p < 0.001). Following 4-week HIIT, the expression of Beclin1, ATG-5, and LAMP-2 improved considerably compared to the DC group (p < 0.001, p < 0.001, and p < 0.05, respectively). In addition, after 4 weeks of curcumin supplementation, the expression levels of ATG-5 and Beclin-1 were significantly improved compared to the DC group (p < 0.001, p < 0.05, respectively). It seems HIIT and curcumin supplementation can be an effective approach for inducing autophagy and improving cardiac function in DCM rats.However, HIIT seems more effective than curcumin in this regard.

**Supplementary Information:**

The online version contains supplementary material available at 10.1186/s13104-023-06295-1.

## Introduction

Diabetic cardiomyopathy (DCM) is considered one of the leading causes of mortality and morbidity in diabetes that occurs in two-thirds of diabetic patients. DCM mainly affects the left ventricular part of the heart without coronary artery disease or arterial hypertension [1–6]. This condition leads to apoptosis of cardiomyocytes, left ventricle dilation, and cardiac dysfunction [7].

Nowadays, a great deal of attention has been paid to an in-depth understanding of autophagy in the etiopathogenesis of DCM. Autophagy seems to be an important player in both cell survival and programmed cell death [7, 8], which is essential as a hemostatic mechanism in the heart to maintain cardiac structure and function [9].

Autophagy-related proteins are key players in this tightly regulated process. The Autophagy Related 1 (ATG1), also known as unc-51 Like Autophagy Activating Kinase 1 (ATG1/ULK1), a pivotal component for the initiation of autophagy, is mainly responsible for receiving cellular stimulating signals and regulating phagophore elongation and autophagosome formation [10]. Autophagy-Related 5 (ATG-5) is another fundamental protein for the formation of autophagic vesicles, playing an important role in autophagy; thus, the knock-down/knock-out process of ATG-5 can lead to the downregulation/suppression of autophagy.

Beclin-1 and lysosome-associated membrane glycoprotein 2 (LAMP-2) are among the key regulators of autophagy. Beclin-1 effectively influences both autophagy and apoptosis; therefore, it can affect the survival and death of cardiomyocytes in the heart [11]. In humans, mutations in the LAMP-2 gene have been reported to cause Danon disease, fatal cardiomyopathy, and myopathy associated with mental retardation. The hearts of mice that are deficient in the LAMP-2 gene show insufficient contractile function and have higher dry heart weight to body weight compared with wild-type mice [12].

In order to unravel the role of autophagy in the pathophysiology of cardiac function during diabetes, researchers have used pharmacological agents and even lifestyle modifications (diet and exercise). High-intensity interval training (HIIT) is a type of exercise that comprises brief bursts of intensive exercise followed by low-intensity rest periods that can cause maximum health during a short period [13, 14]. Little evidence suggests that HIIT leads to cellular process improvement by diminishing the accumulation of dysfunctional proteins and organelles through the promotion of autophagy [15].

Curcumin, a polyphenol found in the Curcuma longa plant, exerts anti-oxidant, anti-inflammatory, anti-cancer, and anti-diabetic properties without considerable adverse effects [16]. Curcumin has been shown to influence autophagy in various types of tumors [17, 18]. Besides, curcumin exerts protective activity in diabetic cardiomyopathy through promoting autophagy-related cell death and ameliorating apoptosis, thus contributing to the improvement of cardiac structure and function [8]. However, there are inconsistent data for curcumin in autophagy regulation in cardiovascular disorders [19, 20].

The main objective of the present study is aimed to clarify whether HIIT and curcumin supplementation affects autophagy pathway in myocardial tissue during diabetic cardiomyopathy in STZ-diabetic rats. Therefore, the expression of autophagy pathway genes (ATG1, ATG5, Beclin1 and LAMP-2) in HIIT and curcumin treatment in control and diabetic rats were evaluated.

## Methods

### Animals

In this experiment, male Wistar rats with 8-week-old and weight of 270 ± 20 g were purchased from the Razi Research Institute (Tehran, Iran). Two animals were placed in a special cage under standard laboratory conditions (temperature 22 ± 3 °C, humidity 45–50%, dark–light cycle 12:12) and were fed with a standard laboratory pellet without restrictions throughout the study. Animals were checked on twice a day for health throughout housing. There were no negative outcomes. one primary (blinded) and one assistant (unblended) investigators were considered in all experiment procedure. While keeping the primary investigator blind, the unblended investigator was in charge of assigning rats and preparing the treatment to be administered during the experiment. The conduct of the experiment and the evaluation of the results were blinded investigator responsibilities. The primary investigator was not blinded for data analysis. one rat served as one experimental unit. All animal experiments were following the National Institutes of Health guide for the care and use of laboratory animals (NIH Publications No. 8023, revised 1978) and approved by Ethics Committee of Shahid Beheshti University of Medical Sciences (IR.SBMU.RETECH.REC.1398.605). There was a determined effort to reduce both the number of animals utilized and their suffering. There were no specified standards for including or excluding animals or data. Due to surgical problems, data from experiments that were early stopped or experienced a prolonged delay (> 7 h) between anesthetic induction and administration of intervention were excluded from analyses. In the diabetic group, if there was no diabetic model (FBG ≥ 126), it was excluded from the study.

### Induction of diabetes and group assigning

Streptozotocin-nicotinamide (STZ-NA) injection (Sigma Aldrich, America) was used to induce diabetes in all rats except for the non-diabetic control (NC) group following 12-h overnight fasting. Single intraperitoneal injection of nicotinamide (120 mg/kg dissolved in normal saline), as well as the intraperitoneal injection of STZ [60 mg/kg dissolved in 0.05 mol citrate buffer (pH = 4.5)], were performed. STZ was administrated 15 min after the administration of nicotinamide. The NC group received the same amount of vehicle (citrate buffer). A glucometer was applied to monitor fasting blood glucose (FBG) 72 h after the injection of STZ-NA to confirm the development of diabetes. Diabetic animals with FBG ≥ 126 mg/dL (7 mM) were chosen for further research. The animals were divided into four groups (n = 6), namely non-diabetic control (NC), diabetic control (DC), diabetes + HIIT (D + HIIT), and diabetes + curcumin (D + CU), One rat was excluded from the NC group due to procedural error, and then 6 rats were randomly selected from the remaining 7 rats. Out of 24 diabetic rats, 4 rats were excluded because they were not diabetic, and then 18 rats were randomly selected from among 20 diabetic rats. The rats in the NC group did not receive curcumin gavage and did not do any exercise program. DC group rats were only induced with diabetes. D + HIIT group rats, in addition to the induction of diabetes, performed HIIT protocol for 4 weeks. D + CU group rats, in addition to inducing diabetes, had gavage of curcumin daily, but did not receive any exercise program. Confounders were controlled by randomizing the order of treatments.

### Exercise program

Five training sessions per week were utilized to familiarize rats with the exercise protocol. In order to become familiar with running on the treadmill, rats were placed comfortably on the treadmill and started training at a very low and consistent speed. The main exercise began 1 week after the familiarization and lasted 4 weeks. The NC, DC, and D + CU groups did not participate in any training but were placed on a treadmill for 5 min five times a week to simulate the same conditions.

HIIT consists of a 3-min warm-up at 30% maximal oxygen uptake (VO2 max), two-minute intervals at 80% VO2max in the first week, 85% VO2 max in the second week, and 90% VO2 max in the third and fourth weeks, with recovery cycles at 30% VO2 max, and a 3-min cool-down at 30% VO2max. In the first and second weeks, there were five repetitions of high-intensity intermittency, and in the third and fourth weeks, there were seven [21]. Running on a rodent treadmill for 4 weeks was regarded as exercise. The training program was continued for 5 days each week, and VO2 max was measured on the 6th day of every 2 weeks.

### Oral administration of Curcumin

The oral administration of curcumin (100 mg/kg as a solution in 4% carboxymethyl oleate solvent) was performed daily in the D + CU group for 4 weeks, and to mimic the stress caused by gavage in all rats, oral administration of water was conducted at the same time on the NC, DC, and D + HIIT groups.

### Echocardiography

The rats were anesthetized 24 h after the last training session and after overnight fasting, by intraperitoneal injection of ketamine (90 mg/kg) and xylazine (10 mg/kg) and the day after euthanized by cardiac puncture using a 27G needle and 3-mL syringe. Echocardiography was employed to determine the ejection fraction (EF %), left ventricular end-diastolic diameter (LVEDD), and left ventricular end-systolic diameter (LVESD). At least three different cardiac cycles were utilized for the measurement of primary echocardiography.

### Real-time polymerase chain reaction (PCR)

In order to assess the mRNA expression, 50 mg of the left ventricular tissue was homogenized in Trizol. The total RNA was isolated from the left ventricular tissue using the RNeasy Micro Kit (Cat No./ID: 74004, Qiagen, Hilden, Germany) according to the manufacturer's protocol. The quantity and quality of the extracted RNA were analyzed using a Nanodrop instrument and gel electrophoresis, respectively. The extracted RNA was reverse-transcribed into the complementary DNA (cDNA) using Transcriptor First Strand cDNA Synthesis Kit (Roche Diagnostics) according to the manufacturer’s protocol. The quantitative RT-PCR was used to evaluate the expression levels of ATG-1, ATG-5, Beclin-1, and LAMP-2 genes using specific primers (Additional file 1: Table S1) by RealQ Plus 2 × Master Mix Green (cat NO./ID: A323402, Amliqon, Denmark). The data obtained from the relative expression of the four genes were normalized against the GAPDH gene.

### Blood analyses

In order to separate serum, blood samples were taken directly from the hearts of animals and poured into the heparin-containing tubes; then centrifuged at 3000 g (Eppendorf made in Germany) at 15 °C for 15 min. The serum levels of fasting blood glucose (FBG) were measured using a quantitative detection kit (Pars Azmoun Co) with a sensitivity of 5 mg/dL. The insulin levels were measured using an ELISA kit (Ultra-Sensitive Rat Insulin ELISA kit, Mercodia, Sweden) according to the manufacturer's protocols.

### Statistical analysis

The obtained values were represented as the means and standard deviation (mean ± SD). The statistical analysis was carried out using Graphpad Prism version 8.00. The Kolmogorov–Simonov test was employed to determine whether the data were normally distributed (k-s). The difference between the groups was analyzed using one-way analysis of variance (ANOVAs), followed by Tukey's post hoc test. The p-value of less than 0.05 was considered statistically significant. ANOVAs is a parametric test is used for data with normally distribution. Normality tests were carried out, which showed multiple variables were not normally distributed, likely because of small values for n. ANOVAs were still used because the central limit thesis states that given enough samples, sample distribution will be normal, regardless of the underlying population distribution.

## Results

### Metabolic parameters of rats

The metabolic parameters of the animals in this research are shown in Table 1. A substantial difference in FBG levels was found between the DC group and NC group, indicating that the induction of diabetes was successfully performed. The serum concentration of FBG was significantly lower in the D + HIIT group compared to the DC group. However, no significant change was detected in FBG between the D + CU and DC groups. Also, the results revealed a significant difference in serum insulin levels between the DC and NC groups. The serum concentration of insulin was significantly increased in the D + HIIT group compared to the DC group. Curcumin had no effect on serum insulin levels by itself, and no significant discrepancies were found between the DC and D + CU groups.

**Table 1:** Metabolic parameters of rats

variable	NC	DC	D + HIIT	D + CU
FBG (mg/dL)	185.5 ± 5.2	576.3 ± 25.5^*^	465.8 ± 47.3^#^	537.3 ± 138.7
Insulin (mg/dL)	2.3 ± 0.14	0.38 ± 0.15*	1.3 ± 0.56^#^	0.66 ± 0.58
FBW (gr)	376.2 ± 13.8	282.5 ± 16.1	267.8 ± 16.6	244 ± 42.1

The values are expressed as mean ± SD; n = 6 for DC group, n = 6 for D + HIIT group and n = 6 for D + CU group. One rat was excluded from NC group due to a procedural error. 4 rats were excluded from diabetic groups because they were not diabetic

*NC* non-diabetic control, *DC* diabetic control, *D* + *HIIT* diabetic-high-intensity interval training, *D* + *CU* diabetic-curcumin, *FBG* fasting blood glucose, *FBW* final body weight

^*^: significantly difference vs. NC

^#^: significantly difference vs. DC

### In-vivo left ventricular function

The findings of the M-mode echocardiograms (Table 2) revealed that in the DC group, the values of left ventricular end-diastolic diameter (LVEDD) and left ventricular end-systolic diameter (LVESD) were significantly higher than in the NC group. LVEDD and LVESD were lower in D + HIIT and D + CU groups in comparison with the DC group; however, only the difference between the D + HIIT group and DC group was statistically significant. In addition, the EF% of the DC group was significantly lower than that of the NC group; however, the percentage of EF was increased after 4 weeks of HIIT protocol and curcumin supplementation. Such an increase was only statistically significant in the D + HIIT group when compared with the DC group.

**Table 2:** Echocardiographic data

variable	NC	DC	D + HIIT	D + CU
LVEDD (mm)	4.6 ± 0.34	7.8 ± 0.42^*^	5.56 ± 0.23^#^	6.8 ± 0.3
LVESD (mm)	2.44 ± 0.26	5.3 ± 0.14^*^	3.26 ± 0.35^#^	4.8 ± 0.23
EF (%)	92.02 ± 1.9	61.96 ± 1.60^*^	71.39 ± 1.31^#^	63.08 ± 2.03

The data are presented as mean ± SD. n = 6 for NC group, n = 6 for DC group, n = 6 for D + HIIT group and n = 6 for D + CU group. One rat was excluded from NC group due to a procedural error. 4 rats were excluded from diabetic groups because they were not diabetic

*NC* non-diabetic control, *DC* diabetic control, *D* + *HIIT* diabetic-high-intensity interval training, *D* + *CU* diabetic-curcumin, *LVEDD* left ventricular end-diastolic diameter, *LVESD* left ventricular end-systolic diameter, *EF* (ejection fraction)

^*^: significantly difference vs. NC

^#^: significantly difference vs. DC

### STZ-induced diabetes inhibits autophagy in cardiomyocytes of diabetic rats

**Fig. 1:**
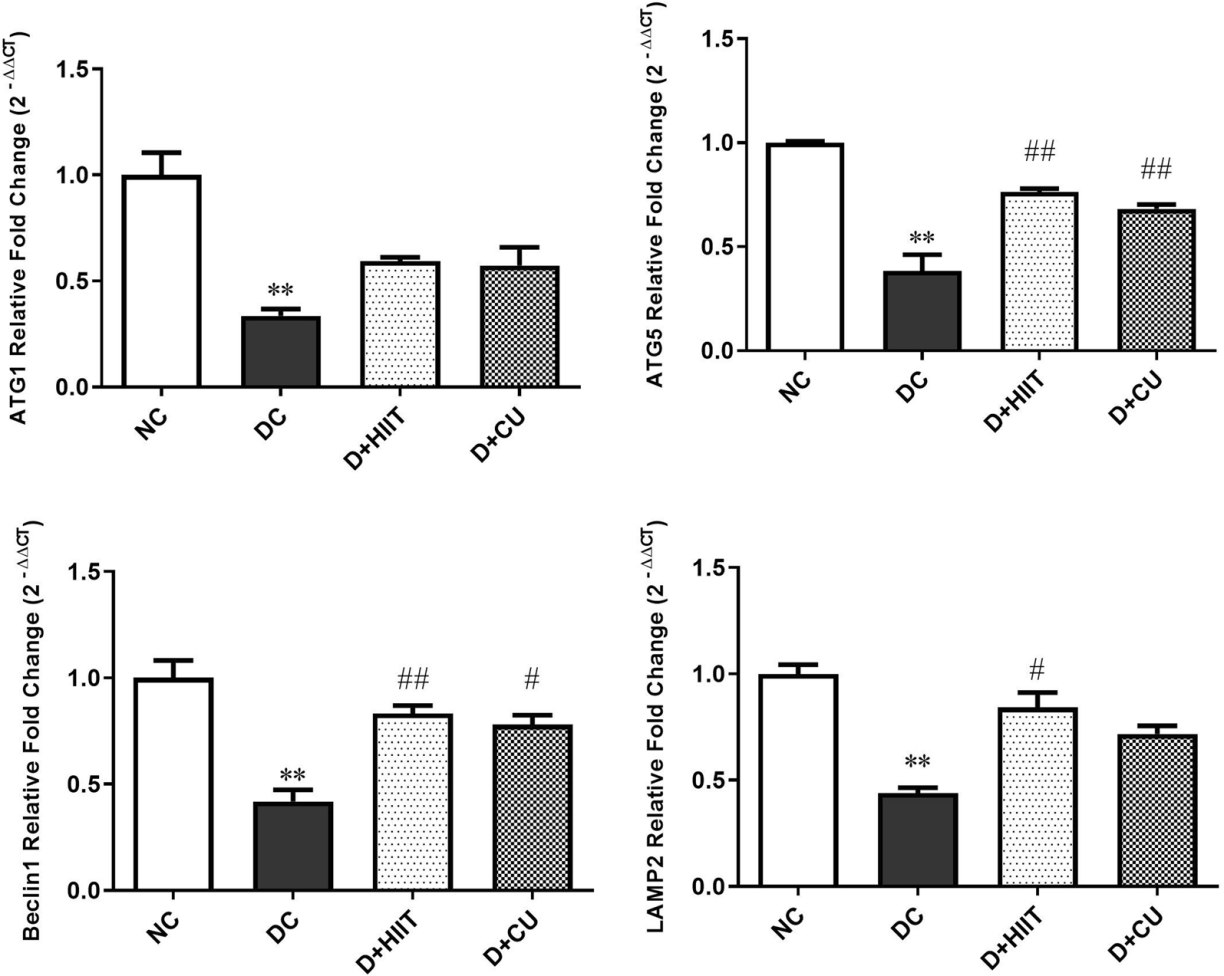
The effects of HIIT and curcumin on gene expression; **a**. ATG-1 expression, **b**. ATG-5 expression, **c**. Beclin-1 expression, **d**. LAMP-2 expression. The obtained values are expressed as mean ± SD. ^*^p < 0.05, ^**^p < 0.001 significant differences vs. NC group, ^#^p < 0.05, ^##^p < 0.001 significant differences vs. DC. Statistical significance was tested using a one-way ANOVA. n = 6 for NC group, n = 6 for DC group, n = 6 for D + HIIT group and n = 6 for D + CU group. One rat was excluded from NC group due to a procedural error. 4 rats were excluded from diabetic groups because they were not diabetic. *NC* non-diabetic control, *DC* diabetic control, *D + HIIT* diabetic-high-intensity interval training, *D + CU* diabetic-curcumin

We first determined whether STZ**-**induced diabetes alters autophagy in cardiomyocytes or not. As shown in Fig. 1, the expression levels of ATG-1, ATG-5, Beclin-1, and LAMP-2 genes were considerably lower in cardiomyocytes of the DC group compared to the NC group (fold change = 0.33; p = 0.0001, fold change = 0.38; p = 0.0001, fold change = 0.41; p = 0.0001, and fold change = 0.43; p = 0.0001, respectively).

### HIIT protocol ameliorates the expression of autophagy genes in the cardiomyocytes of diabetic rats

In order to evaluate a possible beneficial effect of HIIT on the autophagy process in cardiomyocytes, we measured the expression of four genes involved in autophagy. After 4 weeks of HIIT, the expression levels of ATG-5, Beclin-1, and LAMP-2 were significantly higher in cardiomyocytes of the D + HIIT group compared with the DC group (fold change = 1.98; p = 0.0001, fold change = 1.99; p = 0.0001, and fold change = 1.91; p = 0.003 respectively). Also, the expression level of ATG-1 was increased; however, such an increment was not statistically significant (fold change = 1.77; p = 0.102).

### Curcumin ameliorates the expression of autophagy-related genes in cardiomyocytes of diabetic rats

In order to assess the effect of curcumin on autophagy in cardiomyocytes, we measured the expression of four genes involved in autophagy. After 4 weeks of curcumin supplementation, the expression levels of ATG-5 and Beclin-1 genes were significantly elevated in cardiomyocytes of the D + CU group in comparison to the DC group (fold change = 1.77; p = 0.0001, and fold change = 1.86; p = 0.001, respectively). Also, the expression levels of ATG-1 and LAMP-2 genes were increased; however, such an increase was not statistically significant (fold change = 1.70; p = 0.155, and fold change = 1.63; p = 0.06, respectively).

## Discussion

Diabetes has a wide range of complications, the most important of which is cardiovascular disease and DCM [22]. Studies indicated that some positive changes in lifestyle, such as a healthy diet and exercise, can improve abnormalities in the metabolic condition, such as diabetes and its complications, through a variety of mechanisms, such as autophagy [23]. In the present study, we aimed to investigate the impact of HIIT and curcumin, as inducers of autophagy, on cardiomyopathy of STZ-induced diabetic rats.

According to the obtained results, STZ-induced diabetes led to increased serum concentrations of FBG along with reduced insulin levels. After 4 weeks of HIIT and curcumin treatment, FBG and insulin levels were improved in comparison to the DC group, although such improvement was statistically significant only in the D + HIIT group. Furthermore, HIIT had comparable results to curcumin. One plausible reason for increasing glucose levels in diabetic rats can be related to the destruction of pancreatic beta-cells [24]. Therefore, these cells were not able to produce insulin; thus, insulin levels were decreased in the DC group. Consistent with the results of the present study, *Farinha *et al*.* reported an enhanced level of FBG in diabetic patients following exercise [25]. Possible mechanisms for controlling FBG in response to HIIT include increasing glucose uptake in skeletal muscle in insulin-dependent and insulin-independent manners, increasing the number of glucose transporter type 4 (GLUT-4), and enhancing the activity of glycogen synthase [26]. Furthermore, several lines of evidence indicate that HIIT can diminish insulin resistance in animal models and improve glucose metabolism in skeletal muscles by reducing fat mass and enhancing the phosphorylation of protein kinase B (Akt) [14, 27]. Also, studies showed that the amount of insulin required daily was decreased in type 1 diabetic patients 10 weeks after HIIT [25].

*Su *et al*.* revealed that curcumin treatment improved glucose metabolism disorder, enhanced insulin sensitivity, and ameliorated insulin resistance in type II DM rats [28]. Also, *Yu *et al*.* demonstrated that all doses of curcumin could decrease FBG levels in diabetic rats [29]. Preclinical investigations indicate that various dose ranges of curcumin (60–300 mg/kg body weight) can control blood glucose levels through several mechanisms; for instance, curcumin can suppress pro-inflammatory cytokine expression, regulate oxidative stress enzymes, diminish the production of hepatic glucose, inhibit glycogenolysis by the activation of AMP-activated protein kinase (AMPK), and protect pancreatic beta-cells from apoptotic damage during hyperglycemia [30, 31]. A meta-analysis of 11 randomized controlled trials demonstrated that four or more weeks of curcumin /and curcuminoid supplementation could decrease FBG and glycated hemoglobin levels [32]. Besides, *Rashid *et al*.* found that curcumin was effective in increasing insulin levels in diabetic rats [31]. The results of the present study, in line with other research, show an increase in insulin levels and a decrease in FBG after 4 weeks of curcumin treatment, but our data were not statistically significant, which may be due to longer periods of supplementation in other studies [8, 33]. It appears that 4-week HIIT was not enough to affect the levels of FBG and insulin significantly. Moreover, a number of studies suggest that many of the effects of curcumin are dose-dependent [31], with limited bioavailability due to low absorption and rapid elimination [34].

Regarding the impact of curcumin and HIIT on DCM, the left ventricular function of diabetic rats was evaluated using echocardiography. DCM is considered an important complication of diabetes because myocytes in the heart muscle rarely proliferate, and damage to them eventually leads to impaired heart function, decreased myocardial function, and ventricular dilatation [35]. The current study, in line with others, revealed that the values of LVEDD and LVESD were higher while the percentage of EF was lower in the DC group compared with the NC group [36]. Furthermore, our findings indicated that HIIT effectively decreased the values of LVEDD and LVESD, whereas it increased the percentage of EF. In the curcumin-treated group, the same changes were observed, although such alterations were not statistically significant. In agreement with our data, 3-week aerobic training led to the reduced LVEDD and LVESD and an increased percentage of EF in STZ-induced diabetic rats compared to the DC group [37]. Moreover, the significance of exercise in improving cardiac function in diabetic rats has been highlighted [38]. Regarding the role of curcumin treatment in cardiac function, *Yao *et al*.* demonstrated a 3-month treatment with 200 mg/kg of curcumin resulted in a significant reduction in LVESD and an increase in EF% [11]. Also, a 16-week treatment with 100 mg/kg and 200 mg/kg of curcumin in diabetic rats attenuated left ventricular dysfunction induced by diabetes; however, the difference for LVEDD was not statistically significant, while 200 mg/kg of curcumin treatment significantly improved the values of LVESD and EF [31]. These differences might be related to the duration of the treatment course and the dosage of curcumin.

In order to gain insight into the contribution of autophagy to DCM and the effect of curcumin and HIIT, the expression levels of autophagy-related genes, such as ATG-1 (ULK1), Beclin-1, ATG-5, and LAMP-2, were analyzed in the heart tissues of STZ-induced diabetic rats. It is known that autophagy is a defense system that controls the recycling of damaged organelles and protein aggregates, maintaining cellular homeostasis [39]. In order to regulate autophagy in response to energy stress and glucose starvation, the AMPK-mTOR pathway has been recognized as a key mechanism. In mammals, decreased cellular energy generation inhibits mTOR by activating AMPK and phosphorylating the TSC as a result. It is likely that diabetes activates TSC-mTOR signaling by inactivating AMPK, which inhibits the ULK kinase complex and restricts the initiation of autophagy because mTOR negatively regulates autophagy [40].

We found that both HIIT and curcumin restored the expression of key genes in autophagy. However, HIIT had more beneficial effects compared to curcumin. In detail, STZ**-**induced diabetes remarkably reduced the expression of ATG-1, ATG-5, Beclin-1, and LAMP-2. We observed a significant downregulation in the four analyzed genes following the induction of diabetes in the heart tissues of diabetic animals compared to the control group. Importantly, multiple studies reported that the downregulation of autophagy was responsible for a variety of diseases [7, 41, 42].

A difference between the expression levels of ATG-1 and Beclin-1 genes in pancreatic islet cells isolated from multi-organ donors was not statistically significant when the control and diabetic groups were compared; however, the expression of the LAMP-2 gene was markedly decreased [43]. In renal tissues of STZ-induced diabetic mice, the expression of Beclin-1 and ATG-5 proteins remained unchanged, but the expression of the ULK1 protein was downregulated while its mRNA level was decreased slightly, suggesting the downregulation of ULK1 at the post-transcriptional level [44]. ULK1 expression in different cells isolated from the heart tissues (cardiomyocytes, fibroblasts, smooth muscle cells, and endothelial cells) of a mouse model of obesity showed a substantial reduction in the phosphorylation and its protein level just in cardiomyocytes compared to non-obese mice [45]. These contradicted data may result from different types of tissues and diversity in cardiac tissue cells.

Light chain 3 II (LC3-II) is employed for monitoring the process of autophagy and, in this study, was used to analyze cardiomyopathy after 6 months of diabetes induction in OVE26 mice models (a transgenic model of severe early-onset type 1 diabetes). A decrease in the cardiac expression of the LC3-II protein was observed compared to the control group. The reduction of autophagy in ventricular tissues was confirmed using electron micrographic analysis; also, the protein expression of Beclin-1 was diminished in the heart tissue of diabetic mice [39]. The right atrial appendages isolated from diabetic and non-diabetic patients who underwent coronary artery bypass graft surgery demonstrated a significant increase in the protein expression of LC3B-II and Beclin-1. Furthermore, the electron microscopy images confirmed an increment in the number of autophagosomes in the heart tissues of diabetic rats [46]. *Zhang* and colleagues reported a reduction in the protein levels of LC3-II, ATG-5, and ATG-7 in the diabetic group in comparison to the control group [47]. Recently, a study performed on autophagy showed the downregulation of LAMP-2 in the pancreatic beta cells of diabetic and non-diabetic donors [48]. As mentioned earlier, the absence or defect in the autophagy process will be challenging. It has been suggested that proper autophagy induction may reduce cardiomyocyte cell death and cardiomyopathy. Several studies have addressed the role of autophagy in the pathogenesis of heart failure in diabetic patients using gene manipulation, pharmacological intervention, and changes in lifestyle (diet and exercise) [49].

In the present study, the role of HIIT and curcumin, as plausible autophagy regulators, in the transcription levels of key genes in autophagy was investigated. Our findings demonstrated that HIIT had protective effects on DCM in diabetic rats and improved the expression of autophagy-related genes when compared to the DC group. The transcription levels of all genes, except ATG1, were increased significantly.

Anaerobic exercise remarkably increased the expression levels of Beclin1 and LC3-II/LC3-I proteins in skeletal muscle cells of diabetic rat models, which might be attributed to the autophagy activation, thus ameliorating the insulin resistance [50]. Conversely, *Lee* et al. found that 4-week swimming exercise prevented muscle atrophy in diabetic rats by suppressing autophagy and lowering LC3 levels compared to the DC group [51]. According to a study carried out by *Weng *et al*.*, hypoxia inhibited autophagy by lowering ATG1/ULK1, Beclin1, and LC3-II levels and simultaneously boosting apoptosis in CD4 + T lymphocytes obtained from sedentary healthy men, while HIIT enhanced the impaired autophagy and apoptosis processes [52]. The observed inconsistency in exercise effects could be due to the condition of examined tissues. Autophagic flux cannot be determined in vivo and is a limitation for clinical trials. In addition, exercise can be a useful protective factor that tries to maintain normal cellular homeostasis. Curcumin, another independent variable investigated in the present study, significantly improved the transcript levels of Beclin-1 and ATG-5 genes in comparison to the DC group. The upregulation of ATG-1 and LAMP-2 genes was not statistically significant; however, the expression of these two genes was increased. In this regard, *Zhang *et al*.* analyzed podocyte cells and kidney tissue of mice exposed to curcumin for 8 weeks. They revealed that curcumin significantly increased the protein levels of Beclin-1 and ATG-5 and autophagy in podocytes of diabetic mice [53]. *Yao *et al. concluded that LC3-II was decreased in the heart tissues of diabetic mice, but it became normalized following 3-month curcumin treatment. They demonstrated that curcumin protects DCM through AMPK and c-Jun N-terminal kinase 1(JNK1)-mediated phosphorylation of Bcl-2 and Bim and then disassociation of Beclin1-Bcl-2 (or Bim) complexes, and the inhibition of the mammalian target of rapamycin complex 1 (mTORC1) pathway [8]. Furthermore, the modulation of autophagy by curcumin could be an effective strategy for preventing diabetes-related cardiovascular diseases.

Current research has shown that induction of diabetes reduces Beclin-1 levels, which may reflect a decrease in ATG-1 to suppress the initiation of autophagy in cardiomyocytes [54]. Diabetes induction also decreased the expressions of ATG-5 as a component of the ubiquitin-like conjugation system and accordingly diminished the elongation of the phagophore membrane, leading to the limitation of the formation of autophagosomes [55].

Additionally, decreased expression of LAMP-2 induced by diabetes may attenuate the fusion of the mature autophagosomes with cytosolic lysosomes, eventually inhibiting autophagolysosome development [55]. Briefly, STZ-induced diabetes may considerably repress systemic autophagy in cardiomyocytes, thereby suppressing cell initiation, elongation, maturation, and fusion phases. Substantially, a change in Beclin-1 activity through the modulation of its interaction with Bcl-2/XL affects autophagy and apoptosis. On the one hand, diabetes induction suppresses autophagy, while HIIT and curcumin treatment reverse the detrimental effects of diabetes on autophagy and increase the expression of autophagy-related genes. It seems further studies on the autophagy-related function and its association with apoptosis machines are needed to apply what is known for effective DCM treatment. Also human studies should be conducted in the future to confirm our results.

## Conclusions

Overall, we provide evidence for the role of HIIT treatment on FBG and improved insulin levels in STZ-induced diabetic rats. Moreover, the left ventricular function of the animals revealed the protective role of HIIT in DCM. In addition, increasing the transcriptional levels of ATG-5, Beclin-1, and LAMP-2 after HIIT, as well as the expression of Beclin-1, and LAMP-2 genes after curcumin treatment, suggests the protective role of HIIT in DCM. Also, HIIT and curcumin treatment can promote autophagy effectively to enhance cardiac function in DCM; however, HIIT seems to be more effective than curcumin in this regard. Finally, we suggest that to better understanding of HIIT and Curcumin mechanism of action on autophagy pathway in diabetic rat, the mechanistic studies are highly recommended.

### Limitation

The small sample size of the current study is its most important limitation. Also considering that the present study only investigated the expression of genes involved in the autophagy pathway, it should be noted that investigation of autophagy markers at protein levels can confirm the transcript level results.

## Supplementary Information

**Additional file 1: Table S1.** Sequences of rat specific primers used for Real-Time PCR (bp)

## Data Availability

The corresponding author can be reached for a reasonable request for the datasets used and/or analyzed in the current study.

## Abbreviations

DCM: Diabetic cardiomyopathy
HIIT: High-intensity interval training
LAMP-2: Lysosome-associated membrane glycoprotein 2
STZ-NA: Streptozotocin-Nicotinamide
FBG: Fasting blood glucose
EF: Ejection fraction
LVESD: Left ventricular end-systolic diameter
LVEDD: Left ventricular end-diastolic diameter
PCR: Polymerase chain reaction
GLUT-4: Glucose transporter type 4
AMPK: AMP-activated protein kinase
LC3-II: Light chain 3 II
JNK-1: Jun N-terminal kinase 1
MTORC-1: Mammalian target of rapamycin complex 1
