# Supplementary material for: The high-intensity interval training (HIIT) and curcumin supplementation can positively regulate the autophagy pathway in myocardial cells of STZ-induced diabetic rats
Source: BMC Res Notes. 2023 Feb 25;16:21. doi: 10.1186/s13104-023-06295-1 (PMC9960211; doi:10.1186/s13104-023-06295-1)
Supplement: Supplementary file 1 — Additional file 1: Table S1. Sequences of rat specific primers used for Real-Time PCR (bp) [file 13104_2023_6295_MOESM1_ESM.docx]

Additional file table: Sequences of rat specific primers used for Real-Time PCR (bp)

| Target Gene | Sequence 5' 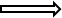 3' |
| --- | --- |
| GAPDH | Forward: TTCTAGAGACAGCCGCATC  Reverse: CAATGTCCACTTTGTCACAAGAG |
| ATG1 | Forward: CTGAAGGAACTAAAGCAC  Reverse: AGGTCTCCACCATTACAATA |
| Beclin1 | Forward: CATTCAGGAACTCACAGC  Reverse: CAATAAATGGCTCCTCT |
| ATG5 | Forward: TCCTGCTCACTGGATG  Reverse: AATCTTCTGCCGCCT |
| LAMP2 | Forward: GGAGATGAATTTCACAATAA  Reverse: TTTGGCACCATTCTTATC |

ATG1: Autophagy-related genes1; ATG5: Autophagy-related genes5; LAMP2: lysosome associated membrane glycoprotein 2 ; GAPDH: Glyceraldehyde-3-Phosphate Dehydrogenase
